# Child Mortality and Child Welfare

**Published:** 1917-05-12

**Authors:** Everitt E. Norton


### CHILD MORTALITY AND CHILD WELFARE.

By EVERITT E. NORTON, M.D., D.P.H.

The widespread interest which has of late been taken in infant welfare work is frequently regarded as the outward sign of a new development. It is, in fact, less a new direction of public health organisation than the corollary, necessarily sequential, to much that has gone before. The routine medical inspection of school children, revealing as it did numerous unrecognised or untended defects amongst school entrants, made evident the need for examination and supervision of children before they attained the school age. The provisions of the Midwives Act, the compulsory notification of infectious diseases (including ophthalmia neonatorum, and recently extended to include measles and German measles), and the supervision of the tuberculous and of those in contact with them, have all been steps in the progress of maternity and infant welfare. We remarked upon this trend of the work of the Public Health Service in the Educational Number of The Hospital of September 2 last. Finally, the Notification of Births Acts, unless they were to be largely sterile, rendered imperative an extension and co-ordination of the work initiated by midwives and their inspectors, health visitors, school and tuberculosis nurses, and voluntary workers. Maternity and child welfare work will not diminish, but will rather increase, the demand upon the hospitals and infirmaries, with the work of which it is likely to prove compatible, even in great towns, to a degree hardly foreseen. Interest in maternity and child welfare has been stimulated indirectly by the war, both through increased attention to the importance of national man-power and through the demand for a wider scope and utilisation of women's work and influence: in so far maternity and child welfare constitute a new branch of public assistance.

Very opportunely is published, as a supplement to the Report of the Medical Officer of the Local Government Board for 1915-16, a Report on Child Mortality, at ages 0-5, in England and Wales, which Report is itself supplemental to four preceding Reports concerning maternity, infancy, and childhood. This collection of statistics and analysis of the incidence and causation of deaths at those ages have been made to show the extent of unnecessary child mortality still occurring, the places in which the need for combating this is most urgent, and the appropriate means for combating it. It is allowed that much saving of child life has already been secured; nevertheless, in some of the urban areas considered the death-rate was three times as high as in some others, and "there is evidently a large mass of preventable mortality."

A striking, almost an appalling, fact in considering the causation of deaths occurring under the age of five years is the great preponderance of deaths from *infections*. Approximately four-fifths of the children dying during the age-period one to five years die from infective diseases; from measles, whooping-cough, diarrhœal diseases, tuberculosis, and forms of pneumonia. "The most striking evidence of possibilities of life-saving during these four years is given by the saving already experienced." Between 1871-75 and 1911-15 there was in the infantile death-rate a decline of 29 per cent.; in the second year of life the corresponding decline was 41 per cent.; in the third year 50 per cent.; in the fourth year 53 per cent.; and in the fifth year 50 per cent. The comparatively small reduction of deaths in the first year after birth is in part ascribable to the fact that but little effect has hitherto been produced on the congenital causes of mortality, which is in itself a sufficient plea for the activities of the maternity and infant welfare centre. The preservation of life in infancy "is more closely a question of intimate personal hygiene than in the next years of life," when the child becomes the prey of the infections. "These are largely controllable; even when not prevented, a fatal result can commonly be obviated if prompt and adequate medical care and nursing are provided"; measles and whooping-cough have been (until the recent efforts initiated by the Local Government Board in the case of the former) "an almost completely neglected field of work for the saving of child life."

The Report deals specially with certain districts and large towns (such as Middlesbrough and St. Helens) where infant and child mortality rates are exceptionally high. The complex causation of this mortality and its indirect, yet definite, relationship to industrial occupations and their accompanying squalor are discussed. "The one outstanding fact is that the centres of excessive child mortality are those in which the chief industries of the country are carried on"; fortunately Sir Arthur Newsholme is of opinion that "this association is not inevitable."

A section of great interest is devoted to the conditions of environment favouring excessive child mortality. "The working-class mother is too often accused of ignorance. . . . This is a facile and unbalanced explanation of the excessive child mortality among the working classes." There is little, if any, difference in degree of ignorance between the wives of wage-earners and the wives of men belonging to other classes. *The difference, apart from the handicap of the former in respect of housing, food supply, and sanitation, in the main is one of ability to secure the assistance required in the various contingencies of maternity and early childhood.*" "The ignorance of the working-class mother is dangerous, because it is associated with relative social helplessness." "Probably more important than actual ignorance is carelessness or fecklessness of mothers"; hand in hand with this go poverty and alcoholic intemperance. The latter is a symptom of social evil as well as its cause, excessive drinking being in part a product of uninteresting surroundings and, more particularly, of domestic discomfort. Even the cinema, with all its vulgarities of picture and poster, has its uses as a rival to the public-house. That poverty (a "complex phenomenon") favours excessive child mortality is a truism; with poverty go bad housing, overcrowding, and uncleanliness, a combination which handicaps from its birth every infant born into it: the working-class mother often is supplied with stale, impoverished milk, may have no pantry, and, except when suckling, has no facilities for the cleanly preparation of her infant's food. "More house pride, and a greater willingness to spend less on ephemeral pleasures and more on domestic comforts are needed. In short, elevation of the standard of living is an indispensable condition of progress. Already the concentration of public opinion in this direction is helping to bring this about."

We commend this most interesting and valuable Report, together with those which preceded it, for careful study by the many who are now directing their attention to the work of maternity and child welfare.

